# Successful Management of Cervical Ectopic Pregnancy with Methotrexate in a Nulliparous Woman: A Case Report

**DOI:** 10.3390/reports8020091

**Published:** 2025-06-07

**Authors:** Franciszek Ługowski, Aleksandra Urban, Joanna Kacperczyk-Bartnik, Ewa Janowska, Jacek Sieńko

**Affiliations:** 2nd Department of Obstetrics and Gynecology, Medical University of Warsaw, 02-091 Warsaw, Poland

**Keywords:** cervical pregnancy, ectopic pregnancy, methotrexate

## Abstract

**Background and Clinical Significance:** Implantation of an embryo in the cervical canal is the rarest location of ectopic pregnancy, as it occurs between 1 in 1000 and 1 in 18,000 pregnancies. Dilation and curettage in previous pregnancies have been identified as risk factors in most cases. Other predisposing factors include pelvic inflammatory disease (PID), prior tubal surgeries, assisted reproductive technologies, as well as the presence of fibroids and intrauterine. Importantly, ectopic pregnancies are the main cause of maternal morbidity and mortality in the first trimester. Given the rarity of cervical ectopic pregnancies (CEPs) and the lack of specific recommendations, clinical data supporting current evidence is of utmost significance. **Case Presentation:** A 29-year-old nulliparous woman presented with spotting from the genital tract and lower abdominal pain persisting for four days. Pregnancy could not be ruled out based on the patient’s medical history. The level of β-Human chorionic gonadotropin (β-HCG) on admission was 1487.99 mIU/mL. The first ultrasonography examination revealed a non-specific imaging appearance suggestive of the presence of cervical mucus. Targeted examination with visualization of the cervical canal revealed a gestational sac measuring 4–5 mm in diameter, containing an embryonic echo. The patient was treated with 84 mg of methotrexate (MTX) i.v. in a 1, 3, 5, 7 scheme along with 0.1 mg/kg calcium folinate i.m. in a 2, 4, 6, 8 scheme prior to curettage. **Conclusions:** A diagnosis of cervical pregnancy cannot be excluded even in the absence of prior risk factors. Methotrexate should be considered a safe and efficient option in the management of CEP. As shown in our case, early detection of CEP is of utmost significance.

## 1. Introduction and Clinical Significance

Implantation of an embryo in the cervical canal is the rarest location of ectopic pregnancy, as it occurs between 1 in 1000 and 1 in 18,000 pregnancies [1]. A cervical ectopic pregnancy (CEP) implanted in the uterine isthmus may develop normally, even until the third trimester. In contrast, one located at the junction of the isthmus and the uterine body may, in some cases, result in the birth of a full-term neonate [2]. Dilation and curettage in previous pregnancies have been identified as risk factors in most cases [3]. Other predisposing factors include pelvic inflammatory disease (PID), prior tubal surgeries, assisted reproductive technologies, as well as the presence of fibroids and intrauterine [3,4,5]. In the course of CEP, the damage to the walls of large blood vessels at the site of embryo implantation, caused by the action of proteolytic enzymes secreted by the trophoblast, may lead to life-threatening hemorrhage [6]. Following implantation, the cervical environment is unable to meet the developmental requirements of the growing ovum due to an incomplete decidual reaction, which impairs normal placental attachment. The cervix, composed primarily of fibrous connective tissue and containing only approximately 15% smooth muscle, offers limited resistance to trophoblastic invasion. As a result, direct infiltration of trophoblastic tissue into the cervical fibromuscular stroma leads to edema, necrosis, hemorrhage, and infiltration by mononuclear inflammatory cells [7]. An inadequate decidual response within the cervical tissue results in abnormal placental adherence, often accompanied by incomplete placental separation and profuse hemorrhage. These pathophysiological features contribute significantly to the elevated intraoperative and postoperative maternal morbidity associated with this condition [7].

Importantly, ectopic pregnancies are the main cause of maternal morbidity and mortality in the first trimester [8]. Given the rarity of CEP and the lack of specific recommendations, clinical data supporting current evidence is of utmost significance.

## 2. Case Presentation

A 29-year-old nulliparous woman presented with spotting from the genital tract and lower abdominal pain persisting for four days. Pregnancy could not be ruled out based on the patient’s medical history. The patient had no prior history of cervical trauma (e.g., surgeries), pregnancy, PID, fibroids, as well as the use of assisted reproductive technologies.

**Figure 1:**
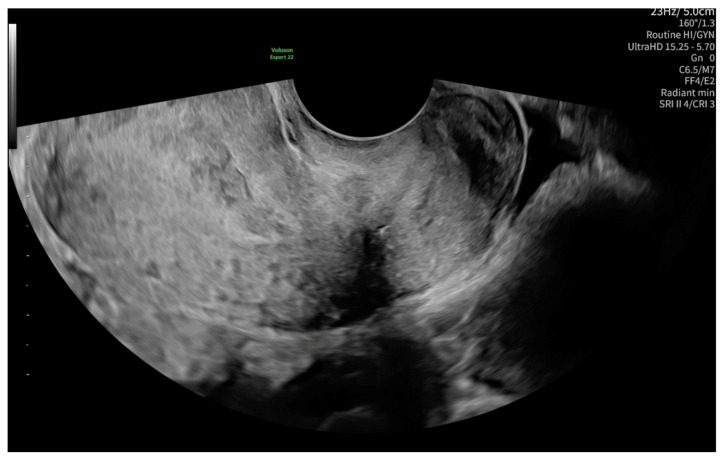
Non-specific ultrasonography image on admission, possibly suggesting the presence of cervical mucus.

The level of β-Human chorionic gonadotropin (β-HCG) on admission was 1487.99 mIU/mL. The first ultrasonography examination revealed a non-specific imaging appearance suggestive of the presence of cervical mucus (Figure 1). No gestational sac was visualized within the uterine cavity. Additionally, no definitive signs of ectopic pregnancy were identified in the adnexal regions. Due to the presence of abdominal pain, the patient was admitted for inpatient observation with a provisional diagnosis of pregnancy of unknown location (PUL).

**Figure 2:**
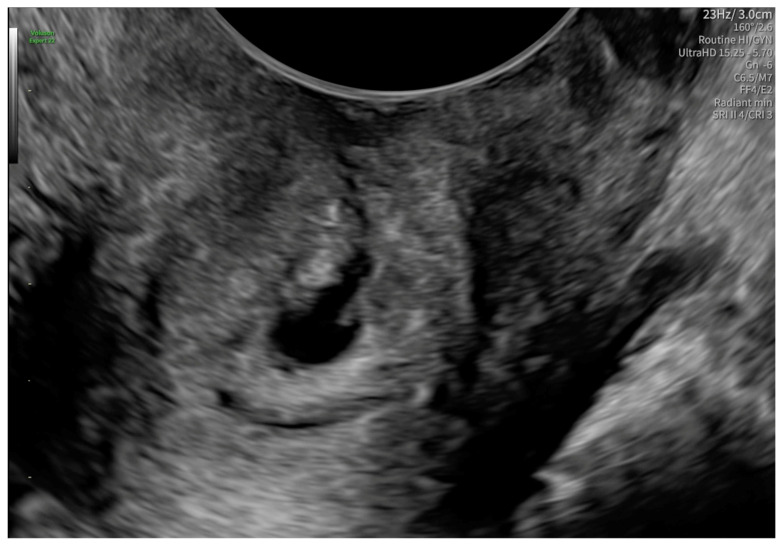
A gestational sac containing an embryonic echo in the cervical canal.

**Figure 3:**
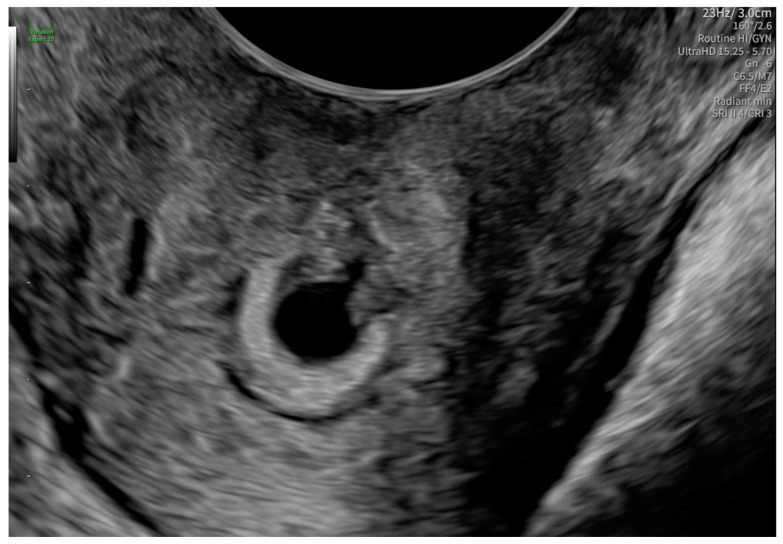
The embryo and yolk sac in a transverse section of the cervical canal.

After 48 h, another transvaginal ultrasonography was performed, revealing cervical canal dilatation greater than previously noted. The cervix was visualized via an ultrasonographic examination with a length of approximately 38 mm. Targeted examination with visualization of the cervical canal (Figure 2) revealed a gestational sac measuring 4–5 mm in diameter, containing an embryonic echo. A single live embryo was identified within the sac, measuring approximately 1.7–1.8 mm in crown-rump length. Fetal heart rate (FHR) of 102 beats per minute was confirmed, and the gestational age corresponded to 6 weeks and 1 day. The trophoblast appeared heterogeneous, with features of linear separation from the posterior wall and scant vascularization of the gestational sac. Figure 3 shows the embryo and yolk sac visualized in a transverse section of the cervical canal.

**Figure 4:**
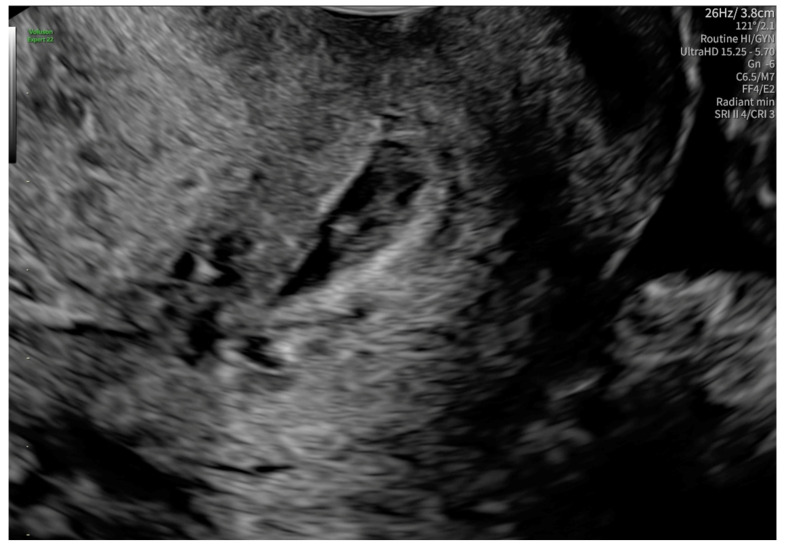
Diagnostic features for CEP: (1) The gestational sac present below the internal os level, (2) a barrel-shaped cervix.

**Figure 5:**
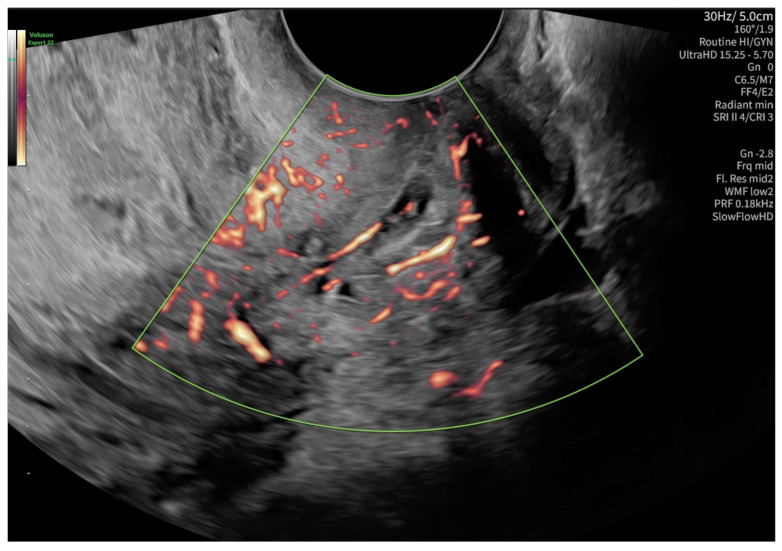
Blood flow around the gestational sac in a color Doppler examination.

**Figure 6:**
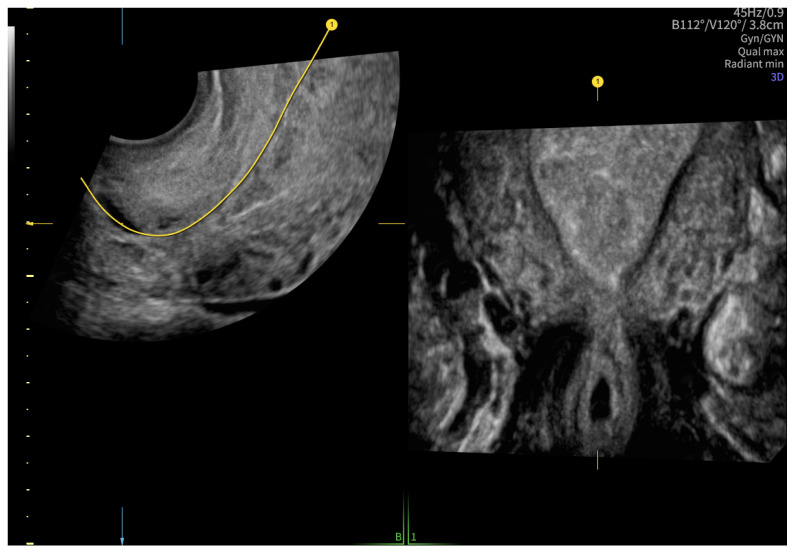
A multiplanar reconstruction of the coronal plane in Omniview mode (GE Voluson).

**Figure 7:**
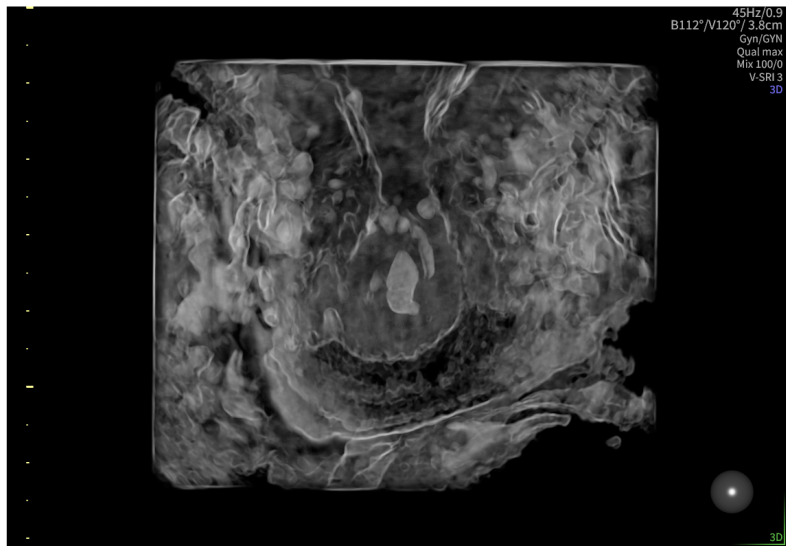
Rendered image in Silhouette mode (GE Voluson) showing mild cervical enlargement with the gestational sac.

According to the Royal College of Obstetricians and Gynecologists (RCOG), based on the presence of the gestational sac below the internal os level, an empty uterus, a barrel-shaped cervix, the absence of “sliding sign”, and blood flow around the gestational sac (Figure 4 and Figure 5), a diagnosis of CEP was made [9]. Figure 6 represents a multiplanar reconstruction of the coronal plane in Omniview mode (GE Voluson), which allows for clear visualization of the gestational sac below the internal os with an empty uterine cavity. Figure 7 illustrates a rendered image in Silhouette mode (GE Voluson) showing mild cervical enlargement with the gestational sac.

Prior to initiating pharmacological therapy, baseline laboratory assessments—including complete blood count, liver enzymes, and renal function tests—were performed and found to be within normal limits. The woman was closely monitored throughout treatment, and no adverse reactions were observed. The patient was treated with 84 mg of methotrexate (MTX) i.v. in a 1, 3, 5, 7 scheme along with 0.1 mg/kg calcium folinate i.m. in a 2, 4, 6, 8 scheme. The treatment was well tolerated. On the next ultrasonographic examination, the pregnancy was nonviable, with no detectable FHR and no vascular flow around the gestational sac. The patient qualified for the curettage of the cervical canal and uterine cavity. The patient was discharged home with a beta-hCG level of 1396.44 mIUml. Histopathological examination of the uterine curettage specimens revealed fragments of compact and spongy decidua with extensive hemorrhagic infiltration, and no chorionic villi or trophoblastic tissue were identified. Moreover, fragments of compact and spongy decidua, as well as chorionic villi of the gestational sac, were identified within the cervical canal, accompanied by extensive hemorrhage. The patient was monitored until serum β-hCG levels declined to undetectable values.

## 3. Discussion

We present a case of successful management of an early CEP with the presence of FHR and a β-HCG value of 1487.99 mIU/mL (the lowest described in the literature regarding CEP), using systemic MTX administration, without hemorrhagic complications. Our decision to use conservative treatment aimed to preserve the patient’s reproductive capability. To our knowledge, previously reported cases of CEP with cardiac activity typically involved higher β-hCG levels, often exceeding 2000 mIU/mL our case, presenting with a value of 1487.99 mIU/mL, represents one of the lowest described in the literature for a viable cervical ectopic pregnancy. Ectopic pregnancy remains the leading cause of maternal morbidity and mortality during the first trimester of pregnancy [8].

The key presenting clinical symptoms typically include mild to moderate vaginal bleeding and cramp-like lower abdominal pain, although the latter is not invariably present. On examination, the cervix may exhibit soft enlargement due to an intracervical mass, with a closed internal os and an open external os [10,11]. Hemorrhage triggered by manipulation or cervical dilation necessitates prompt intervention [11].

Although no standardized treatment guidelines exist for CEP, medical management, particularly with methotrexate, is widely considered the first-line approach for hemodynamically stable patients during the first trimester. The selection of therapy is multifactorial and depends not only on gestational age, fetal cardiac activity, and serum β-hCG concentration, but also on the extent of cervical vascularization and bleeding risk, the patient’s hemodynamic status, and the desire to preserve future fertility. Early diagnosis is critical, as timely intervention allows for conservative management and minimizes the likelihood of invasive procedures, such as hysterectomy [12]. Advanced GA is associated with greater morbidity and a higher risk of hysterectomy [13]. Emergent literature data suggest that MTX’s anti-trophoblastic effects are efficient in CEP; therefore, we administered it to our patient prior to curettage [13,14]. Other treatment options include transvaginal injection of MTX, misoprostol, uterine artery embolization, hysteroscopy, or hysterectomy [13,14,15]. Notably, surgical methods of management are associated with a high failure rate and should be reserved for those women suffering life-threatening bleeding [9].

MTX therapy is commonly associated with adverse effects such as vaginal spotting and gastrointestinal symptoms, including nausea, vomiting, and diarrhea [16]. Abdominal pain may occur within 2–3 days post-treatment. In the absence of clinical signs suggestive of tubal rupture, this can generally be managed conservatively [16]. Patients are advised to avoid the use of non-steroidal, anti-inflammatory drugs, as these agents may interfere with MTX efficacy. Additionally, the use of substances that could obscure symptoms of tubal rupture, such as opioids, alcohol, or other analgesics, should be avoided. Activities that may elevate the risk of rupture, including vaginal intercourse, are also discouraged [17]. Given the teratogenic potential of MTX, effective contraception is recommended for at least three months following treatment, although this guidance is based on limited empirical evidence [18].

The success rate of MTX in resolving ectopic pregnancies without the need for surgical intervention is reported to range between 70% and 95%, with lower efficacy observed in patients presenting with elevated baseline serum β-hCG concentrations [16,17]. However, recent meta-analyses have yielded inconsistent findings regarding the efficacy and safety profiles of various MTX treatment protocols [19,20,21]. These discrepancies underscore the need for further high-quality research to better inform clinical decision-making.

Alternative pharmacologic strategies have also been explored. For instance, the combination of oral gefitinib administered over seven days with a single intramuscular dose of MTX has shown promise in the management of stable tubal EPs, effectively obviating the need for surgical intervention [22]. While these preliminary findings are encouraging, validation through randomized controlled trials is required before such regimens can be adopted into standard clinical practice. Alternative management strategies for CEP include local methotrexate injection, uterine artery embolization, hysteroscopic resection, and, in severe cases, hysterectomy [23,24,25,26]. However, many of these methods carry a higher risk of bleeding, are more invasive, or may compromise future fertility. In comparison, systemic methotrexate, when used in carefully selected patients, offers a non-invasive, fertility-preserving option with a favorable safety profile, as demonstrated in our case. Given the absence of formal guidelines for cervical ectopic pregnancy, clinical decision-making must rely on case-based evidence and expert consensus. This case adds to the growing body of literature supporting conservative, fertility-preserving management in stable patients with early CEP.

What distinguishes this case is the exceptionally low β-hCG level (1487.99 mIU/mL) at the time of diagnosis despite the presence of embryonic cardiac activity, a combination rarely documented in the literature. Additionally, the patient was nulliparous and had no identifiable risk factors, further underscoring the importance of high clinical suspicion and individualized management.

## 4. Conclusions

This patient’s presentation highlights that the diagnosis of cervical pregnancy cannot be excluded even in the absence of prior risk factors. Methotrexate should be considered a safe and efficient option in the management of CEP. The presented report underscores the importance of individualized treatment planning based on hemodynamic stability, gestational parameters, and fertility considerations. As shown in our case, early detection of CEP is of utmost significance, as we have described a successful treatment of a patient with the lowest β-HCG value noted in the literature.

## Data Availability

The data presented in this study are available on request from the corresponding author, as the European Union and Polish Law restrict the publication of patients’ sensitive data.
